# Intraoperative Embolization during Inferior Vena Cava Tumor Thrombectomy for Renal Cell Carcinoma

**DOI:** 10.15586/jkcvhl.v10i4.299

**Published:** 2023-12-30

**Authors:** Samantha A. Roberts, Divyaam Satija, Henry Gold, Mina S. Makary, Jing G. Wang, Eric A. Singer, Tasha Posid, Nahush A. Mokadam, Shawn Dason

**Affiliations:** 1 Division of Urologic Oncology, The Ohio State University Comprehensive Cancer Center, Columbus, OH;; 2 Wright State University Boonshoft School of Medicine, Dayton, OH;; 3 Division of Cardiac Surgery, The Ohio State University Wexner Medical Center, Columbus, OH;; 4 Division of Vascular and Interventional Radiology, Department of Radiology, The Ohio State University Wexner Medical Center, Columbus, OH;; 5 Division of Pulmonary and Critical Care Medicine, Department of Internal Medicine, The Ohio State University Wexner Medical Center, Columbus, OH

**Keywords:** embolization, intraoperative, RCC, thrombectomy, tumor

## Abstract

Intraoperative tumor thrombus embolization is a potentially lethal complication during inferior vena cava (IVC) thrombectomy for renal cell carcinoma (RCC). Intraoperative embolization is uncommonly encountered because IVC thrombectomy surgical technique is focused on avoiding this complication. Nonetheless, early recognition of embolization is essential so that emergent management can be instituted. When available, cardiopulmonary bypass (CPB) and embolectomy should be considered the gold standard for the management of intraoperative embolization. Several novel endovascular techniques are also available for selective use. We present the case of a 71-year-old female with a right renal mass and level II (retrohepatic) IVC tumor thrombus. During cytoreductive nephrectomy and IVC thrombectomy, tumor embolization was diagnosed during a period of hypotension based on transesophageal echocardiographic finding of new thrombus within the right atrium. This prompted sternotomy, CPB, and pulmonary artery embolectomy. The patient survived this embolization event and has a complete response to systemic therapy 9 months postoperatively. This case serves as the framework for a discussion on management considerations surrounding intraoperative embolization during IVC thrombectomy.

## Introduction

Inferior vena cava (IVC) tumor thrombus is found in 10% of patients undergoing nephrectomy for renal cell carcinoma (RCC) (1). The presence of an IVC tumor thrombus poses a number of important considerations during nephrectomy (2). Foremost amongst these is the potential for embolization of the tumor thrombus during the operation. We present a case and discussion focused on the management of acute tumor thrombus embolism.

## Case Report

**Figure 1:**
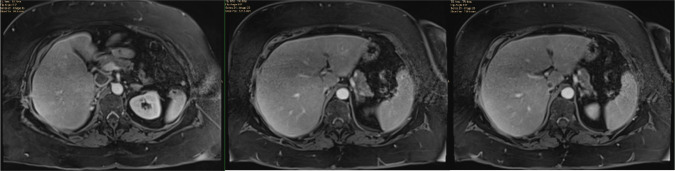
Pre-operative MRI showing tumor thrombus extending into the IVC below the major hepatic veins.

A 71-year-old female was assessed at our center for a new diagnosis of metastatic RCC. She presented to an outside hospital with hematuria and was found to have a 10 cm renal mass with tumor thrombus extending into the vena cava below the main hepatic veins (Mayo level II tumor thrombus) (Figure 1). Staging workup revealed multiple lung nodules up to 1.5 cm on CT which were biopsy proven to be metastatic clear cell RCC. Her medical history was notable for a pulmonary embolism (PE) diagnosed 9 months prior to presentation for which she completed a 6-month course of oral anticoagulation.

On presentation, her hemoglobin was 9 g/dL (lower limit of normal 11.4 g/dL), platelets were 422 K/µL (upper limit of normal 400 K/µL), neutrophil count was 8.46 K/µL (upper limit of normal 7.28 K/µL), calcium was 9.3 (normal), and ECOG performance status was 1. It was likely that she would require systemic therapy within the next year. These findings were all consistent with IMDC poor risk disease. She was symptomatic with hematuria that required recent hospitalization and transfusion.

Lengthy shared decision-making was conducted to decide on the first steps of her mRCC management. The evidence and rationale for upfront systemic therapy vs. cytoreductive nephrectomy were discussed at length (3, 4). Decision-making was influenced by her hematuria requiring recent hospital admission and transfusion, her need to restart anticoagulation given the prior PE in the setting of malignancy, her good baseline health and performance status, and limited asymptomatic extrarenal disease at presentation. After shared decision-making, a plan was made to pursue cytoreductive nephrectomy and IVC thrombectomy.

Transesophageal echocardiography (TEE) at the beginning of the operative procedure showed normal heart function with no RV dilation or evidence of emboli. TEE access was maintained throughout the case.

**Figure 2:**
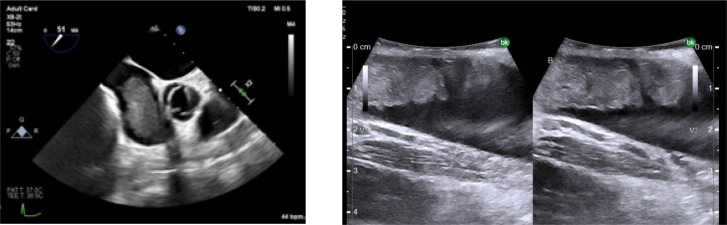
Pre-operative TEE showing friable thrombus tip.

A standard chevron and retroperitoneal exposure were performed (5). Due to the friable tip of the thrombus (Figure 2), a decision was made to approach this as a level III tumor thrombus with suprahepatic control to avoid the extensive IVC manipulation required to release significant portions of the caudate and obtain an infrahepatic clamp (5). Suprahepatic circumferential control of the IVC was obtained along with the left renal vein and infrarenal IVC.

**Figure 3:**
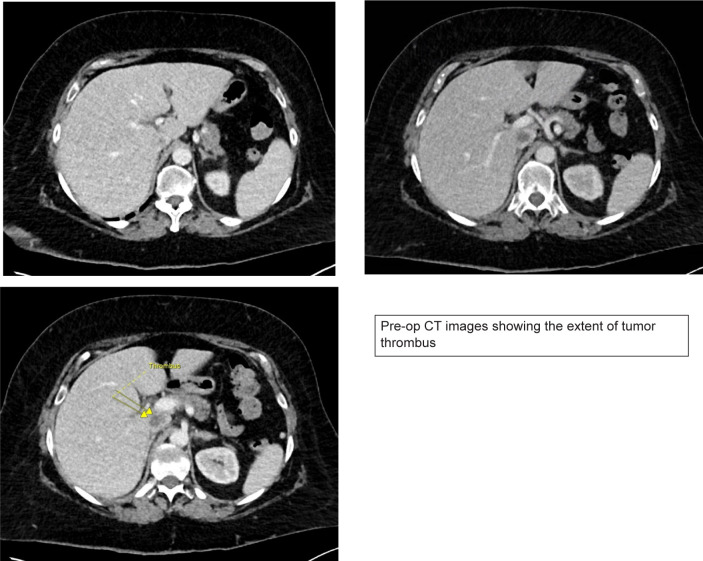
Intraoperative TEE showing tumor thrombus in the right atrium.

After IVC control was obtained, attempts were made to ligate the right renal artery in the interaortocaval space (5). There was some difficulty visualizing the right renal artery due to tumor-related desmoplastic reaction and the patient’s obesity. During this dissection, the patient became hypotensive with systolic pressures in the 70s. This prompted the urologic surgeon (SD) to request TEE review, which revealed tumor thrombus filling most of the right atrium (Figure 3).

Although the originally noted hypotension responded well to resuscitation, it was evident that hemodynamic collapse was imminent given the size of the thrombus filling the right atrium. The patient was administered 25,000 units of intravenous heparin. Cardiac surgery and interventional radiology were called for emergent assistance.

While awaiting assistance, a decision was made to emergently complete the nephrectomy and IVC thrombectomy. This was performed because: (i) most of the setup had been completed and it was felt that this could be completed expeditiously, (ii) significant heparin had been administered and the bleeding risk of the partially dissected kidney with significant neovascularization was felt to be high, (iii) there was a risk for further embolization with *in situ* tumor thrombus, and (iv) it would take time for assistance to arrive and set up.

The nephrectomy and residual IVC thrombectomy were completed in the 10 minutes it took for assistance to arrive. A vascular stapler was used to control the area where the right renal artery should lie behind the right renal vein. The infrarenal IVC, suprahepatic IVC, porta hepatis, and left veins were clamped, vena cavotomy was made, and residual tumor thrombus was extracted with nephrectomy (5). While the IVC was open, brief attempts were made to release the suprahepatic clamp and extract the right atrial thrombus (RAT) with an Yankauer suction under echocardiographic guidance. These attempts were unsuccessful due to thrombus size. Following IVC closure with 4-0 prolene, the abdomen was packed with laparotomy pads with the intention of a second-look laparotomy following stabilization.

As the procedure above was completed, multidisciplinary assistance had arrived including cardiac surgery, interventional radiology, and multiple cardiac anesthesiologists. TEE showed significant intracardiac tumor thrombus now obstructing the right ventricular outflow and hemodynamic instability was worsening. Options for management were discussed including cardiopulmonary bypass (CPB) and embolectomy vs. endovascular extraction. Given the instability, thrombus size, and operating room location, it was felt that survival was most likely with CPB and embolectomy. A conference was held with the patient’s family and the surgical team to confirm that this would be within the patient’s goals of care.

The patient underwent a median sternotomy, providing good exposure of the thoracic cavity. As the pericardium was opened, it became evident that the tumor had traversed into the pulmonary artery by this point. Once CPB was established, a longitudinal incision was made in the main pulmonary artery, and further extended into the left pulmonary artery, allowing excellent exposure of the tumor thrombus. Due to the substantial size of the tumor, it could not be extracted as a single specimen. Therefore, it was removed partially in a morcellated fashion, allowing for complete removal, while preserving integrity of the surrounding structures. There was no visual evidence of remaining embolus in the main, right or left pulmonary artery following pulmonary artery embolectomy. CPB was weaned without significant difficulty and TEE showed good biventricular function with no sonographic evidence of remaining tumor embolus. The sternotomy was closed, and the patient was transferred to the cardiovascular intensive care unit.

The patient remained stable over the next 24 hours allowing for return to the OR for removal of lap pads and permanent abdominal closure. There was no evidence of hemorrhage; however, the caval reconstruction was reinforced due to expeditious repair the previous day. There was also repair of a small right hemidiaphragm defect that likely resulted from *en bloc* diaphragm resection with nephrectomy.

She was extubated and therapeutic anticoagulation resumed the day after secondary closure. Issues during her hospital stay included hypotension requiring vasopressors for 48 hours attributed to vasoplegia, mobility difficulties, oxygen requirement, and urinary retention. She was discharged 2 weeks after surgery to a rehabilitation center. Pathology from the pulmonary artery embolectomy revealed clear cell RCC. Her pulmonary disease progressed radiologically for which she was started on nivolumab monotherapy 3 weeks after the operation. She was readmitted once in the postoperative period with respiratory difficulties requiring diuresis and thoracentesis. During routine re-staging before systemic therapy was initiated, she was noted to have an asymptomatic solitary 4 mm brain lesion. Stereotactic body radiotherapy (SBRT) was used to treat this brain metastasis. Given her response to nivolumab alone in the early perioperative period, a tyrosine kinase inhibitor was not added. She currently has a complete radiologic response to single-agent nivolumab and remains alive at 9-month follow-up. Patient consent was obtained, and institutional review board waiver was applicable for quality improvement data review.

## Discussion

Intraoperative tumor embolization is a unique complication that can occur during IVC thrombectomy for RCC. This is often a fatal complication and mortality was only avoided in this case with early recognition and emergent pulmonary artery embolectomy.

The most specific study on intraoperative tumor embolization originates from the University of California, Los Angeles (6). Review of their 282 IVC thrombectomy cases identified a case series of five patients undergoing nephrectomy with IVC thrombectomy complicated by intraoperative thrombus embolization. The authors describe a tumor embolization rate of 1.8% and a mortality rate associated with embolization of 60%. Tumor embolization occurred during kidney or liver mobilization and presented with sudden cardiac arrest or hemodynamic instability. Four out of five patients underwent operative management of the thrombus embolus, and one out of four operatively managed patients survived. The patient who received medical management was more hemodynamically stable at the time of embolism detection than the patients who were managed operatively and also survived. An additional series from the Cleveland Clinic describes tumor embolization requiring pulmonary artery embolectomy occurring in four patients (5.^3^% incidence for level II–IV thrombi in their series) with three of four patients surviving (25% mortality rate) (7). Other series have described similar findings (8, 9).

### Role of TEE

This case highlights the importance of TEE and multidisciplinary pre-operative planning for Level II–IV tumor thrombus in RCC. TEE findings can guide surgical approach by reliably identifying the cephalad extension of the tumor thrombus and thrombus characteristics, like adherence, fragility, consistency, and mobility (10, 11). Pre-operative discussions with cardiac anesthesia are essential for coordinating TEE. Intraoperative TEE is particularly useful for:

1. Determining the cephalad extent of Level IV (supradiaphragmatic) tumor thrombi. This can influence surgical management. In the event of an infraatrial Level IV thrombus seen on TEE, an infraatrial clamp rather than CPB may be used.
2. In the retrohepatic and supradiaphragmatic IVC segments that are difficult to visualize, TEE may also be useful to ensure complete resection of tumor thrombus.
3. TEE is useful in monitoring volume status and cardiac function, especially during cross-clamping of the IVC, which is associated with decreased central venous pressure and up to 50% reduction in cardiac output (11, 12).
4. As evidenced in this case, intraoperative TEE is critical for the prompt recognition of tumor embolization.

### Surgical Pulmonary Embolectomy

Surgical resection of the tumor embolus is the cornerstone of tumor emboli management (8). While pulmonary artery embolectomy is rarely used in contemporary practice for bland pulmonary emboli due to alternatives like anticoagulation and endovascular thrombectomy techniques, it remains the gold standard for the management of intraoperative tumor embolization.

Modern surgical pulmonary embolectomies have historical roots in the Trendelenburg procedure. First described by Friedrich Trendelenburg in 1908, this approach consisted of hemisternotomy over the left sternal border and a mini-thoracotomy at the left second intercostal space (13). The resulting “T”-shaped incision was used to visualize the main pulmonary artery and the ascending aorta (13). Both great vessels would be temporarily occluded using an encircling band, while a small pulmonary arteriotomy was made for emboli extraction (13). Once control of the arteriotomy was achieved using a tangentially applied clamp, the great vessel occlusion was released (13). Despite this innovative approach, it was only first successful in 1924 and subsequent success was rarely reproducible. The advent of CPB in 1953, however, revolutionized the surgical management of acute massive PE, increasing survival of surgical embolectomy to up to 89% in 2002 (14).

Modern surgical approach to PE relies on placing the patient on CPB after completing a full median sternotomy and entering the pericardium (14). While some studies, such as the one conducted by Aklog et al. (14), employ a transverse arteriotomy post-CPB considering its lower risk of stenosis, our case presented a unique scenario. Given the need for enhanced visualization due to the tumor’s intricate trajectory, a longitudinal arteriotomy was deemed necessary to ensure a thorough tumor removal and mitigate potential complications. We believe that the choice between transverse vs. longitudinal arteriotomy should be tailored to the specific characteristics of each case, weighing the benefits of improved visualization against the risk of stenosis.

### IR management of cardiac tumors

Although embolectomy is the gold standard for intraoperative tumor embolization in patients with hemodynamic compromise, significant advances have been made in endovascular techniques that are also relevant to select patients. These approaches can be particularly useful when CPB and embolectomy are not available or when detection of the embolus occurs post- or pre-operatively and the patient is in a stable condition.

Minimally invasive management of RAT or PE can be performed with vacuum-assisted thrombectomy (VAT). VAT relies on aspiration for emboli removal and has been shown to be successful in the removal of RAT and PE (15–19). The AngioVac system (Vortex Medical; Ontario, Canada) is highly effective in RAT removal with a success rate of approximately 68% and partial success rate of 11% (15). The AngioVac system has an elevated risk of complications when used for PE management, including 11.1% risk of right ventricular perforation (15). These discrepancies in success, however, may be attributed to the studied patient population with PE having more hemodynamic instability than the patients with RAT when undergoing VAT (15).

The emergence of newer thromboaspiration devices, like the FlowTriever (Inari Medical; Irvine, CA) and the Indigo Aspiration System (Penumbra Inc; Alameda, CA), offers less invasive alternatives. Traditionally, a minimally invasive approach to pulmonary thromboemboli involves manually fragmenting the thrombus with continuous rotation of a pigtail catheter, thereby increasing the surface area of the thrombus to optimize anticoagulation disintegration of the clot (16). In the case of tumor emboli, however, this approach would have limited benefit. The FlowTriever and Indigo Aspiration system have been shown to be effective in reducing right ventricular burden in the management of PE with low incidence of major adverse events and mortality rates (17–19). Limited literature is available on the utility of these devices in RAT management. Nezami et al. were able to successfully retrieve an intraoperative RAT using the FlowTriever system (20). Similarly, Peterson et al. demonstrated the successful removal of RAT using the Indigo Aspiration System, indicating that the FlowTriever or the Indigo Aspiration System could be employed for successful RAT and PE outcomes (21).

### Management algorithm

In the event of intraoperative embolization, we propose that management can be determined by three factors: hemodynamic status, suspicion of tumor thrombus, and location of the embolus. Patients with high suspicion of tumor embolus who are hemodynamically unstable should be managed using CPB and surgical embolectomy, regardless of the embolus location. However, if CPB is not available or if the patient is hemodynamically stable, VAT can be considered. If VAT is pursued, the location of the thrombus can be used to determine which technology would be best suited. The AngioVac system has been shown to be highly effective in acute RAT removal and should be first employed (15). If the embolus is in the pulmonary arteries, then FlowTriever and Indigo Aspiration System have been shown to be effective with less complications (15, 18–21).

If surgical or endovascular options are unavailable, transfer to a tertiary center should be considered. Anticoagulation alone could be considered for the stable patient with limited embolic burden.

If bland (rather than tumor) embolus is suspected in the unstable patient with PE, standard interdisciplinary PE management is warranted. Medical management with anticoagulation is generally sufficient for the hemodynamically stable patient.

## Conclusion

Intraoperative embolization is an important consideration during nephrectomy and IVC thrombectomy for RCC. Multidisciplinary management is essential for survival (22).

**Figure:**
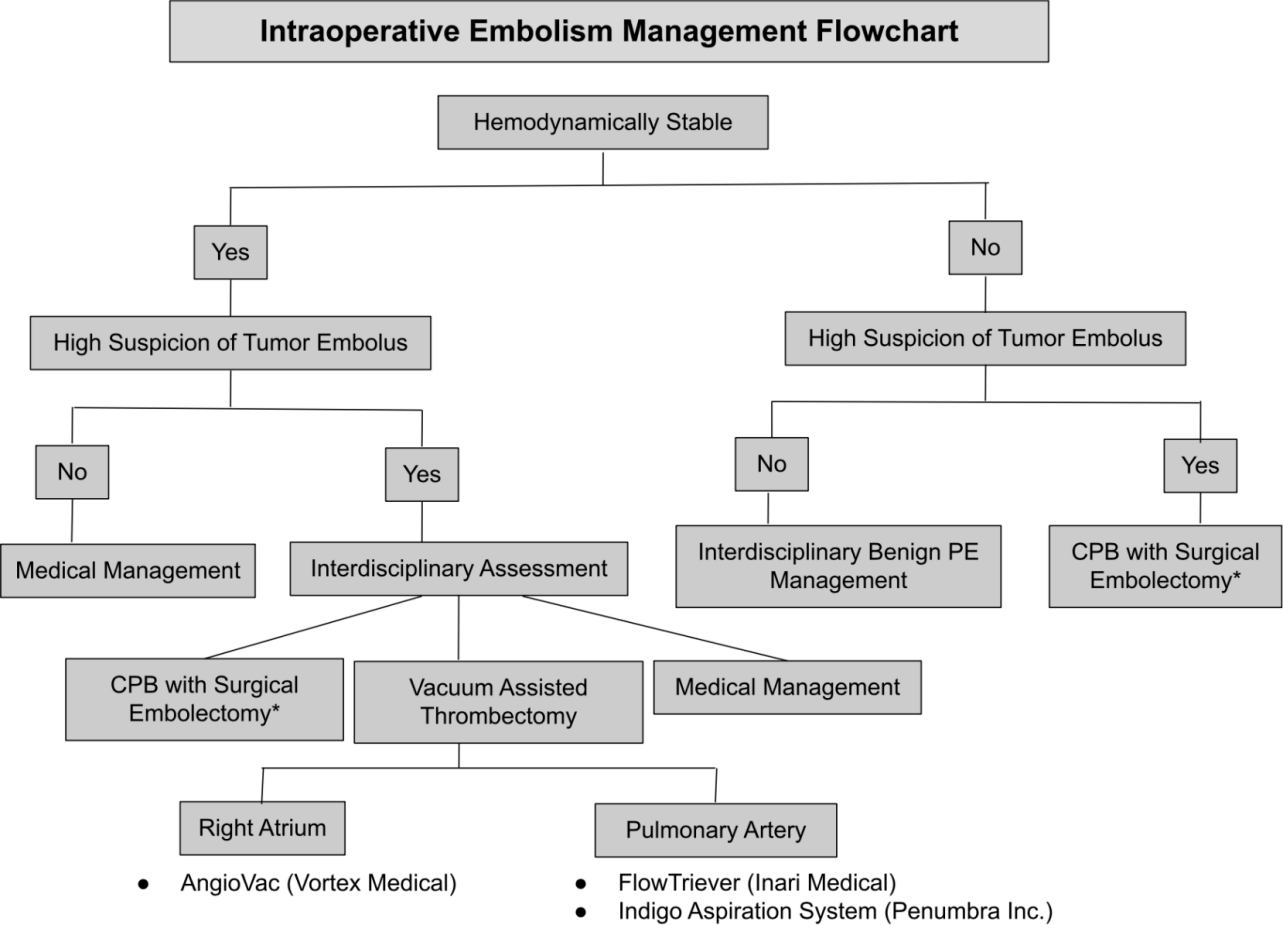
*If CPB is unavailable, then consider the VAT approaches listed under embolus location. If no CPB or operative options are available, proceed to heparinization.
